# Correlations of Neuropsychological and Metabolic Brain Changes in Parkinson's Disease and Other α-Synucleinopathies

**DOI:** 10.3389/fneur.2019.01204

**Published:** 2019-11-14

**Authors:** Maja Trošt, Matej Perovnik, Zvezdan Pirtošek

**Affiliations:** ^1^Department for Neurology, University Medical Center Ljubljana, Ljubljana, Slovenia; ^2^Faculty of Medicine, University of Ljubljana, Ljubljana, Slovenia; ^3^Department for Nuclear Medicine, University Medical Center Ljubljana, Ljubljana, Slovenia

**Keywords:** Parkinson's disease, cognitive impairment, α-synucleinopathies, dementia with Lewy bodies, multiple system atrophy, [^18^F]-fluorodeoxyglucose positron emission tomography (FDG-PET)

## Abstract

Cognitive impairment is a common feature in Parkinson's disease (PD) and other α-synucleinopathies as 80% of PD patients develop dementia within 20 years. Early cognitive changes in PD patients present as a dysexecutive syndrome, broadly characterized as a disruption of the fronto-striatal dopamine network. Cognitive deficits in other domains (recognition memory, attention processes and visuospatial abilities) become apparent with the progression of PD and development of dementia. In dementia with Lewy bodies (DLB) the cognitive impairment develops early or even precedes parkinsonism and it is more pronounced in visuospatial skills and memory. Cognitive impairment in the rarer α-synucleinopathies (multiple system atrophy and pure autonomic failure) is less well studied. Metabolic brain imaging with positron emission tomography and [^18^F]-fluorodeoxyglucose (FDG-PET) is a well-established diagnostic method in neurodegenerative diseases, including dementias. Changes in glucose metabolism precede those seen on structural magnetic resonance imaging (MRI). Reduction in glucose metabolism and atrophy have been suggested to represent consecutive changes of neurodegeneration and are linked to specific cognitive disorders (e.g., dysexecutive syndrome, memory impairment, visuospatial deficits etc.). Advances in the statistical analysis of FDG-PET images enabling a network analysis broadened our understanding of neurodegenerative brain processes. A specific cognitive pattern related to PD was identified by applying voxel-based network modeling approach. The magnitude of this pattern correlated significantly with patients' cognitive skills. Specific metabolic brain changes were observed also in patients with DLB as well as in a prodromal phase of α-synucleinopathy: REM sleep behavior disorder. Metabolic brain imaging with FDG-PET is a reliable biomarker of neurodegenerative brain diseases throughout their course, precisely reflecting their topographic distribution, stage and functional impact.

## Introduction

Parkinson's disease (PD) is a chronic neurodegenerative disease affecting 2–3% of the population older than 65 years. It is primarily a movement disorder characterized by bradykinesia, rigidity, postural impairment, and resting tremor. Its neuropathologic hallmarks are degeneration of substantia nigra and intracellular aggregation of α-synuclein (1). Since James Parkinson's *An Essay on the Shaking Palsy* (2), which still remains largely valid, new knowledge has been gained, particularly on non-motor symptoms, some of which may precede motor signs by decades (3). Cognitive decline, which was not described by James Parkinson, is one of the most debilitating non-motor symptoms and it may drastically decrease patient's and caregiver's quality of life. It is now recognized that the full spectrum of cognitive decline, ranging from subjective cognitive decline (SCD) through mild cognitive impairment (MCI) to dementia can be observed in PD patients (4).

Furthermore, cognitive impairment is important for diagnosis and differential diagnosis among α-synucleinopathies, since some are strongly associated with dementia [PD and dementia with Lewy bodies (DLB)] and others not [multiple system atrophy (MSA) and pure autonomic failure (PAF)]. Although underlying pathology (α-synuclein) is the same in all-aforementioned conditions, its topography, pathophysiology and clinical presentation of cognitive impairment may differ.

Cognitive impairment is a common feature of PD and based on different criteria and thresholds, 10–40% of PD patients have MCI (PD-MCI) at the time of the diagnosis (5–11). Among the PD patients with normal cognition at baseline almost 50% develop MCI within 6 years (12), however around 20% of patients with PD-MCI revert to normal cognition after a year (13). Additionally, subtle cognitive decline, presenting as a decline in the processing speed and executive functions as well as a mild decrease in Mini-Mental State Examination (MMSE) score may even precede PD diagnosis for up to 7 years (14). SCD, which is a risk factor for Alzheimer's dementia (AD), is believed to also precede MCI in PD, and SCD in PD remains an active research topic (4).

Current diagnostic criteria proposed by Movement Disorder Society define PD-MCI as a gradual decline of cognition, not causing a significant impact on a patient's everyday functioning. It is defined by clinical, cognitive, and functional criteria (15). PD-MCI is a heterogeneous disorder and it can be either amnestic or non-amnestic (16). PD-MCI correlates with an increased risk of developing dementia (12, 13, 17). PD dementia (PDD) causes cognitive changes in more than one domain and affects the subject's day-to-day functioning (18). It is a common feature of the disease and, if patients live long enough, it occurs in almost 80% of them within 20 years from the initial diagnosis (19). On the other hand, patients who develop dementia prior or within 1 year of first motor signs of parkinsonism are diagnosed with DLB (20). While DLB and PDD are both characterized by similar pathology and cognitive impairments, e.g., executive function, attention (21, 22), DLB patients perform worse on visuospatial and memory tests (23). Their cognitive decline is faster with more severe fluctuations and their survival-time is shorter (24, 25).

MSA is a less common α-synucleinopathy characterized by autonomic failure, cerebellar and parkinsonian signs and one third of patients develop frontal-lobe dysfunction (26, 27). A minority of MSA patients also develop dementia syndrome, which has a rather heterogeneous clinical presentation (28–30). α-synucleinopathies are commonly preceded by prodromal conditions, such as idiopathic rapid eye movement (REM) sleep behavior disorder (RBD) or, rarer, PAF. RBD, not an α-synucleinopathy by itself (31), is characterized by lack of muscle atonia during REM sleep and it has been shown that RBD patients perform worse on neuropsychological tests compared to healthy controls (32, 33). A big majority of RBD patients also develop α-synucleinopathies; PD, DLB, or MSA (34–37). In PD patients, RBD correlates with a higher prevalence of PD-MCI and its presence predicts cognitive decline at follow-up (38–41). RBD may therefore offer an insight into the development of dementia at its preclinical phase and be an excellent target for disease-modifying interventions. However, reliable and objective biomarkers of progression of cognitive impairment and conversion to dementia are still under development (42). PAF is characterized by α-synuclein inclusions in peripheral autonomic nervous system and consequential autonomic symptoms (43). PAF, similarly to RBD, often progresses to various α-synucleinopathies, although its relation to later cognitive decline is still unclear (44).

The etiology of cognitive impairment in PD can be divided into two partially overlapping orthogonal patterns, according to the dual syndrome hypothesis (45). Cognitive changes in planning ability, working memory and executive function (dysexecutive syndrome) in PD-MCI patients arise due to disruption of the fronto-striatal dopamine network, which is mainly caused and driven by the depletion of striatal dopamine transmission, rather than by primary frontal dysfunction (46). Executive functions are also closely correlated with the mesocortical dopamine system, which arises in the ventral tegmental area and projects to the neocortical areas and whose hyperactivity may act compensatorily in the early PD when only the fronto-striatal system is impaired. There is some evidence that disruption of both dopaminergic systems is necessary for the development of dysexecutive syndrome (45, 46). Disruption of the noradrenergic and cholinergic system further contributes to the executive dysfunction (46). On the other hand, patients who mostly suffer from deficits in visuo-spatial function and semantic fluency, already early in PD course, have marked posterior cortical and temporal lobe dysfunction (45). It has been shown that the latter subgroup of patients develops dementia more rapidly (47). Attention, visuoperceptual, and memory deficits also correlate with the neurodegeneration of the cholinergic nucleus basalis of Meynert and a consequential disruption of the posterior cholinergic network (46).

[^18^F]-fluorodeoxyglucose positron emission tomography (FDG-PET) is a well-established diagnostic method in early and differential diagnosis of neurodegenerative brain diseases, including dementia (48). FDG enters the cells via glucose transporter, where it is metabolized and stays trapped in the cell, and where ^18^F decays (49). Although it is still under debate whether FDG signal mainly reflects neuronal or astrocytic glucose metabolism (50, 51), it is still thought to be a direct measurement of synaptic activity (52) and is in close correlation with cerebral blood flow (53, 54). It was shown that impaired glucose metabolism antecedes atrophy in Parkinson's and Alzheimer's disease and that these two processes represent consecutive changes of neurodegeneration (55, 56), making FDG-PET an excellent candidate for an early disease stage biomarker. As demonstrated by a recent meta-analysis, functional brain abnormalities detected with FDG-PET scan, are more consistently and reliably observed in PD patients than are the structural changes detected with voxel-based morphometry magnetic resonance imaging (MRI) (57). Although the topography of hypo/hypermetabolic changes is thought to be specific for different neurodegenerative disorders (58, 59), FDG-PET information in the clinical setting is only regarded as supportive or not supportive of the diagnostic hypothesis and it is recommended to be always used in addition to clinical and neuropsychological assessment (48).

The last few decades brought us enormous progress in both image acquisition techniques and subsequent FDG-PET image analysis methods. These have broadened our understanding of disease processes in neurodegenerative brain diseases through defining regional disease-related metabolic changes as well as by investigating their long-range consequences on the spatial connectivity and whole brain metabolic changes (60).

Although international guidelines suggest the use of quantitative techniques for aiding the interpretation of brain FDG studies (61, 62), in clinical setting visual assessment of FDG-PET images may still be deemed appropriate, depending on the individual, local procedures. Visual assessment, however, harbor limitation measured by inter-rater variability, mainly depending on the expertise and experience of the reader (63). Assessment by visual reading can be improved by the use of various statistical mapping approaches (64, 65). The use of automated semi-quantification methods is advised by the European Association of Nuclear Medicine and the European Academy of Neurology for increased accuracy of image reading (66). The most widely accepted methods are based on mass univariate testing, such as statistical parametric mapping (SPM) (67, 68). For clinical evaluation, it has been established to apply SPM for voxelwise comparison of e.g., regional FDG uptake of a single patient's image with the age matched control group images, preferentially acquired at the same site. In this approach, each voxel is evaluated independently, without an a-priori hypothesis and voxel clusters that are statistically significantly different, after correction for multiple comparisons, between the patient's and control group's images can be identified and mapped onto an anatomical atlas or an individual, structural MRI for further interpretation (68). Other statistical approaches include multivariate analyses, such as scaled subprofile model/principal component analysis (SSM/PCA). When properly applied, this method can be used to generate specific disease-related patterns (58, 69, 70). Pattern's expression can be prospectively measured and quantified from the individual scans with the Topographic Profile Rating (TPR) analysis (70). For all the methods, however, a basic knowledge of both, disease characteristics and statistical procedures is needed for proper interpretation of the results.

As most neurodegenerative brain syndromes manifest with a range of cognitive impairments, neuropsychological assessments represent the gold standard of its assessment. Metabolic imaging may significantly contribute to our understanding of functional anatomy and pathophysiological underpinnings of cognitive impairments.

The aim of this review was driven by two basic questions: (i) do neuropsychological data in PD and related α-synucleinopathies correlate with metabolic neuroimaging data and (ii) do these neuroimaging data reveal correlates of impaired cognition already in syndromes that are known to predict evolution into PD, DLB, and MSA.

## Methods

A literature search on PubMed was performed using terms: “cognitive,” “cognition” or “neuropsychological” and “Parkinson,” “Parkinson's,” “dementia with Lewy bodies,” “DLB,” “LBD,” “PDD,” “Multiple system Atrophy” or “MSA” and “Fluorodeoxyglucose,” “Fluoro-deoxyglucose,” “FDG,” “hypometabolic” and variations, or “hypermetabolic” and variations. Additionally, for our second aim, we included search terms “REM Sleep Behavior Disorder,” “RBD,” “Pure autonomic failure” or “PAF.” Two hundred sixty-four articles were found and analyzed. Only original research articles relevant to the aforementioned rationales and pertaining to human studies, written in the English language, published up to June 2019 were included in this review. Case reports, interventional studies, comparisons with non-α-synucleinopathies, studies investigating non-cognitive signs, etc. were not taken into account.

## Parkinson's Disease

We reviewed studies investigating MCI in PD, its progression to PDD and studies specifically addressing the correlation between metabolic changes and neuropsychological deficits.

Already in 1992, Peppard et al. described that cognitive decline in PD is accompanied by changes in brain glucose metabolism (71). More recent studies focused on cognitive decline in specific stages of PD. In comparison with healthy control (HC) participants, it was shown that PD-MCI patient exhibit regional glucose hypometabolism in temporo-parieto-occipital regions (72–74). The same pattern of hypometabolism, although to a lesser extent, is seen when comparing PD-MCI with PD patients having normal cognition (72, 73, 75, 76), marking posterior, presumably cholinergic disruption. Although, when Lyoo et al. divided PD patients into MCI subgroups, the single domain amnestic subgroup exhibited no differences in comparison to HC (73). Lack of differences may be accounted to small sample size (*n* = 12) and topographic heterogeneity of hypometabolic brain changes in amnestic MCI subgroup. Rather inconsistent are also findings of the frontal metabolic changes in PD-MCI in comparison to HC. Some studies report frontal hypometabolism (72, 73, 76) and the others frontal hypermetabolism in paracentral lobule (75) in PD-MCI patients. This inconsistency can be explained to some degree by selective focusing on hypometabolic changes in some studies and thus not paying attention to hypermetabolic ones. The hypermetabolic changes however previously rose some controversy regarding their meaning (77–80). Recent results (81–83) show that relative hypermetabolism is a true (compensatory) feature of cognitive changes in neurodegenerative diseases and not just a side-effect of normalization. Furthermore, heterogeneity of PD-MCI sample (84), different image reconstruction algorithms (85) or the selection of a comparison group could also be a source of confounding results. It is also a possibility that mild frontal hypometabolic changes were not seen in a small sample studies due to stringent statistical thresholds. Studies investigating the progression of cognitive decline showed that extensive parietal and occipital hypometabolic changes can predict the development of PDD in PD-MCI patients (65, 86–88) and also in PD patients with normal cognition (88, 89). Changes in glucose metabolism were also shown to be correlated with global cognition changes and other neuropsychological tests (90, 91).

Only few studies investigated the correlation of regional metabolic brain changes with cognitive dysfunction detected by neuropsychological tests.

Deficits of executive functions correlated with frontal hypometabolism in some studies (92, 93), but not in the others (94–96). Furthermore, disruption of the striatal dopaminergic system, shown with [^18^F]-6-fluorodopa PET imaging, correlated with executive dysfunction in patients with preserved metabolism in the frontal cortex (97), with uncertain explanation. The effect of anti-parkinsonian medication on metabolic changes, which may influence fronto-striatal dopaminergic network (98), has not been thoroughly addressed as of yet. Hypometabolism in parietal and temporal cortices more consistently correlated with executive dysfunction (92–96). Attention deficits, regarded by some as an executive dysfunction (99), correlate with hypometabolism in the frontal cortex (95), precuneus and parietal cortex (94) and with the hypermetabolism in the putamen, parahippocampal gyrus, inferior frontal lobule, paracentral lobule, and hippocampus (94). Posterior cholinergic neurodegeneration, marked by the initial decline in visuospatial functioning and followed by memory impairment, can be detected by glucose metabolism changes, too. The former deficits correlate with the occipito-parietal, temporal and precuneal hypometabolic changes (94, 96), but also with the putaminal and parahippocampal hypermetabolism (94). Memory deficits correlated with the temporal and parietal hypometabolism (92, 94, 95). However, both, hypo- and hypermetabolism in the posterior cingulate cortex were found to correlate with memory deficits (94, 96).

**Figure 1:**
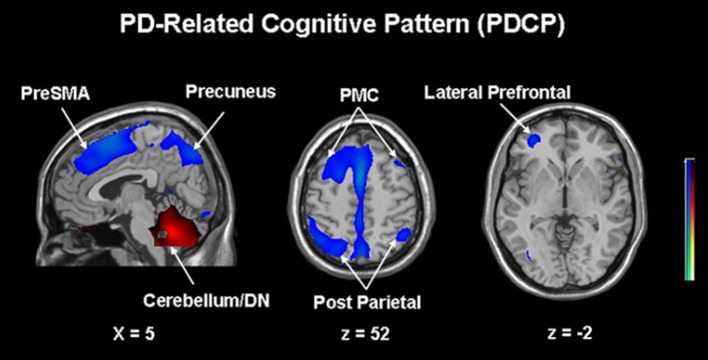
Parkinson's disease-related cognitive pattern (PDCP) identified by scaled subprofile model/principal component analysis (SSM/PCA) from a group of 15 non-demented PD patients. PDCP is characterized by bilateral hypometabolism in the supplementary motor area (preSMA), precuneus, the dorsal premotor cortex (PMC), inferior parietal lobule and left prefrontal region and relative increases in the cerebellar vermis and dentate nuclei (DN). Voxels showing metabolic increases are color-coded red and those showing metabolic decreases are color-coded blue. Reprinted with permission from Huang et al. (101).

**Figure 2:**
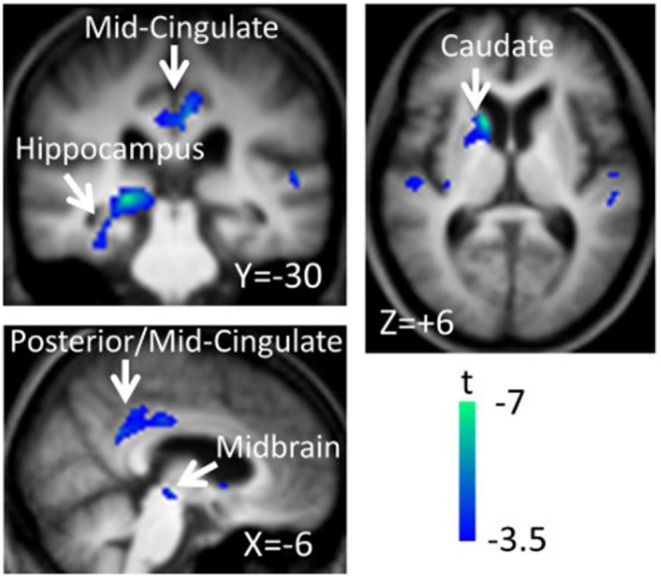
Parkinson's disease dementia (PDD)-cognition related pattern identified by scaled subprofile model/principal component analysis (SSM/PCA) from a group of 18 demented PD patients. Pattern significantly correlated with Mini-Mental State Exam Score (*r* = −0.483, *p* = 0.042). PDD-related cognition pattern is characterized by hypometabolism in the left caudate nucleus, middle and posterior cingulate gyri, temporal regions, amygdala, hippocampus and midbrain and no metabolic increases were found. Voxels showing metabolic decreases are color-coded blue. Reprinted with permission from Ko et al. (111).

The next paragraph focuses on metabolic network analyzes in cognitive changes in PD. A specific regional metabolic covariation pattern, associated with poor performance on tests of executive control and attentional control of working memory, was identified in non-demented PD patients using the region of interest-based SSM/PCA analysis. It was characterized by increased metabolic activity in the left pallidum and mediodorsal thalamus associated with decreased metabolic activity bilaterally in the ventromedial frontal regions, striatum and in the left hippocampal gyrus (100). Later, a voxel-based adaptation of SSM/PCA was used to identify a specific spatial covariance pattern associated with cognitive functions in PD patients, termed PD-related cognitive pattern (PDCP) (Figure 1). PDCP was identified and validated in two groups of non-demented PD patients. Both patient groups were relatively young, 58.6 and 58.8 years, respectively and had a high MMSE score of 28.3 and 28.1, respectively. The magnitude of PDCP expression correlated with a test of executive function (Trail Making Test B), Symbol Modality Test, memory functioning (California Verbal Learning Test) and test of visuospatial function (Hooper Visual Organization Test). This cognitive pattern did not correlate with patients' motor impairment. PDCP is characterized by bilateral hypometabolism in the supplementary motor area, precuneus, the dorsal premotor cortex, inferior parietal lobule and left prefrontal region and relative increases in the cerebellar vermis and dentate nuclei. Furthermore, PDCP has shown to be a robust metabolic indicator of cognitive decline in PD, as its scores were stable in a group of patients which was scanned twice over two months (101). PDCP encompass both posterior and frontal changes as well as compensatory hypermetabolic changes and therefore present a reliable, objective biomarker of cognitive decline. Similarly, another specific brain metabolic network, termed PD-related pattern (PDRP), which correlates with the severity of motor symptoms, was identified prior to PDCP (102). PDCP was later also identified in two different cohorts and the results significantly correlated with the original one (103, 104). Both newly-derived PDCPs also correlated with neuropsychological tests of executive functions (103, 104). Two longitudinal studies showed that the expression of PDCP increases over time, but its expression lags behind the expression of the motor function related PDRP (105, 106). This is in consistence with clinical findings in PD, where motor symptoms precede significant cognitive changes (1). Furthermore, it was shown that PDCP expression is in correlation with the worsening of cognitive impairment (107) and with the loss of dopaminergic input in the anterior striatum, particularly in the caudate nucleus, as shown with dopamine transporter imaging ([^18^F]-fluoropropyl-β-CIT PET and [^18^F]-fluorodihydroxyphenylalanine (FDOPA) PET) (108, 109). Hypermetabolic cerebellar changes, once argued to be an artifact (77), have recently been proven a true feature of cognitive decline representing a compensatory activation of cognitive networks including the cerebropontocerebellar tract (81). Since original PDCP was identified in non-demented patients, further studies explored its relationship to other dementia syndromes. Mattis et al. applied TPR algorithm to calculate individual's expression of PDCP and showed that it is not expressed in patients with AD (110). Ko et al. identified a different and specific brain metabolic pattern of cognitive decline in PDD (Figure 2). PDD cognition-related pattern was characterized by decreased metabolism in the left caudate nucleus, middle and posterior cingulate gyri, temporal regions, amygdala, hippocampus and midbrain and no metabolic increases were found. This pattern was identified in a group of patients with PDD with an average age of 70.7 years and MMSE score 16.2. PDD cognition-related pattern negatively correlated with MMSE score (111). Its topography is similar but not identical to the PDCP identified in non-demented PD patients. Similar but not the same statistical methods were recently used on resting-state functional MRI data (rs-fMRI). Independent component analysis identified rs-fMRI PDCP (fPDCP), which was topographically similar to its FDG-PET derived counterpart. fPDCP was characterized by reduced regional activity in the precuneus, medial parietal cortex, medial prefrontal and supplementary regions, thalamus and inferior parietal cortex (112). For a more detailed explanation of PDCP, we refer the reader to a recently published review article (113). The detailed table with studies investigating neuropsychological changes in correlation with glucose metabolism in Parkinson's disease is available as a supplementary material (Table S1). Future studies may be warranted to investigate the PDCP or fPDCP's ability to detect individuals with worse prognosis of cognitive decline.

## Multiple System Atrophy

In general studies investigating metabolic changes in correlation to neuropsychological findings are lacking in MSA. A few studies conducted so far showed consistent hypometabolic changes in frontal cortex, striatum and cerebellum, the latter only in MSA-cerebellar or mixed-type (114–116). As cognitive impairment progresses in multiple domains, hypometabolic changes were not unexpectedly observed in temporo-parietal regions (115). Another group found no correlation between glucose metabolism and MMSE scores (114), but MMSE is probably not sensitive enough to detect cognitive changes in MSA and more detailed neuropsychological testing may be more appropriate in MSA patients.

## Dementia With Lewy Bodies

In DLB metabolic brain changes are found in temporo-parietal, posterior cingulate, frontal association and primary visual cortex (117–119). A small study (11 patients) from Fujishiro et al. showed that hypometabolism in primary visual cortex can predict DLB in non-demented individuals (120). Furthermore, Sala et al. showed disruption of posterior cortical networks in DLB patients, especially in the primary visual network (121). Specific disease-related changes in glucose metabolism can be seen in DLB patients even before the development of distinct clinical picture. Early detection of such changes may thus significantly shorten the time to correct diagnosis (122).

Only a few studies directly compared PDD to DLB which are clinically and pathologically similar syndromes and even these with conflicting results. Hypometabolic changes were found in the anterior cingulate cortex in DLB patients in one study (123), while another one found no differences in metabolic topography between DLB and PDD (124). Abovementioned PDD cognition-related pattern was expressed also in DLB patients, though the difference in expression was of borderline statistical significance and there is a need for further exploration of network differences between these two disorders (111).

Awareness of memory impairment was studied on a group of DLB patients and it was found to correlate with hypometabolism in the posterior cingulate cortices bilaterally and the right orbitofrontal cortex (125). Recently, a large multicenter study of 171 DLB patients used whole-brain parcellation approach guided by PCA and followed by linear regression analysis to identify metabolic patterns correlated to the core features of DLB and cognitive decline. Cognitive fluctuations were found to be associated with hypometabolism in bilateral occipital cortices and with hypermetabolism in the parietal lobe. Furthermore, a sensitivity map of the disease severity (measured by MMSE score) was constructed and the posterior cingulate cortex was identified as a region most closely correlated with the decline in MMSE (126).

## Rapid Eye Movement Sleep Behavior Disorder and Pure Autonomic Failure

RBD is a prodromal phase of Lewy body disorders and even at this prodromal stage metabolic changes pointing toward PD, DLB, or MSA can be seen (40, 127, 128). SSM/PCA network analysis was used to identify RBD-related pattern (RBDRP). It was characterized by increased metabolic activity in the pons, thalamus, precentral gyrus, supplementary motor area, medial frontal gyrus, hippocampus, parahippocampal gyrus, supramarginal and inferior temporal gyrus, and posterior cerebellar tonsils and associated with decreased metabolic activity in the occipital regions, midbrain (red nucleus) and superior/middle temporal gyrus. Interestingly, expression of RBDRP was high in early-stage, but not in late stage PD (129), which hints toward the change in predominant networks involved in diseases as it progresses from RBD to PD. RBDRP was later identified also in a different cohort of patients (130, 131). Surprisingly, the univariate analysis does not reveal increased metabolism in thalamus, supplementary motor area and extensive cerebellar changes (132), meaning that multivariate analyses may detect subtler brain activity changes compared to the univariate ones. The RBDRP's expression was found to be significantly higher in PD-MCI patients than in PD patients without cognitive decline (130) and it correlated with tests of executive function (131). RBDRP's expression may therefore be related to worse cognitive status in individual PD patients. To the best of our knowledge RBDRP's expression has not been investigated in DLB as of yet. But interestingly, there are metabolic differences between DLB patients with and without RBD, with the former having more extensive metabolic decreases throughout the whole brain (119). There are no published studies investigating neuropsychological and metabolic changes in pure autonomic failure.

## Conclusions and Future Perspectives

Neuropsychological changes are among the most common and debilitating non-motor symptoms in PD, they are essential for DLB diagnosis and seem to be present in RBD already. FDG-PET brain imaging is a valuable tool to study the underlying mechanism of cognitive dysfunction in PD and other α-synucleinopathies. We may conclude that neuropsychological data in PD and related α-synucleinopathies correlate with metabolic neuroimaging data, although there are some controversial findings in these metabolic-cognitive correlations, which should be further addressed. Similarly so, the differentiation between the causal and compensatory metabolic changes in these disorders.

Although the studies investigating neuropsychological changes and glucose metabolism in PD related α-synucleinopathies are not many and a majority of them are retrospective, results do reveal correlates of impaired cognition already in RBD and those in most cases predicts the evolution into α-synucleinopathies.

Further research effort should be directed toward prospective follow-up studies of these syndromes from their prodromal stages, to be able to capture subtle metabolic brain changes, already before dementia arises. These may become valuable biomarkers of disease progression and/or conversion to dementia. Recent methodological advances brought forth various objective and quantifiable covariance patterns, which consistently and reliably correlate with cognitive changes and may already predict the disease progression. However, future research is needed to validate these disease-related patterns in larger, multicentric cohorts taking into account an important need for standardization of imaging reconstruction and analysis protocols (133).

Furthermore, topics beyond the scope of this review—neuropsychiatric changes, such as apathy, anxiety and depression, which are common in patients with neurodegenerative brain disorders, need to be addressed as well as their impact on patients' cognitive functions and brain metabolism.

Last but not least, new analytical tools, such as deep learning, that are currently under development may be able to pick up complex neurological circuits involved in cognitive changes in combination with imaging of the neurotransmitter changes that underlie the brain activity changes in PD and other α-synucleinopathies.

## Author Contributions

MT and ZP conceptualized the paper and provided inputs and edits. MP performed the systematic search of the literature and wrote the first draft, created the table, and finalized the paper.

### Conflict of Interest

The authors declare that the research was conducted in the absence of any commercial or financial relationships that could be construed as a potential conflict of interest.
